# EPHB6 and testosterone in concert regulate epinephrine release by adrenal gland chromaffin cells

**DOI:** 10.1038/s41598-018-19215-2

**Published:** 2018-01-16

**Authors:** Yujia Wang, Wei Shi, Alexandre Blanchette, Junzheng Peng, Shijie Qi, Hongyu Luo, Jonathan Ledoux, Jiangping Wu

**Affiliations:** 1 Research Centre, Centre hospitalier de l’Université de Montréal (CRCHUM), Montreal, Quebec H2X 0A9 Canada; 2 Montreal Heart Institute, Montreal, Quebec H1T 1C8 Canada; 3 Nephrology Department, CHUM, Montreal, Quebec H2L 4M1 Canada; 4 The Children’s Hospital, Zhejiang University School of Medicine, Hangzhou, Zhejiang 310003 China

## Abstract

Erythropoietin-producing human hepatocellular receptor (EPH) B6 (EPHB6) is a member of the receptor tyrosine kinase family. We previously demonstrated that EPHB6 knockout reduces catecholamine secretion in male but not female mice, and castration reverses this phenotype. We showed here that male EPHB6 knockout adrenal gland chromaffin cells presented reduced acetylcholine-triggered Ca^2+^ influx. Such reduction depended on the non-genomic effect of testosterone. Increased large conductance calcium-activated potassium channel current densities were recorded in adrenal gland chromaffin cells from male EPHB6 knockout mice but not from castrated knockout or female knockout mice. Blocking of the large conductance calcium-activated potassium channel in adrenal gland chromaffin cells from male knockout mice corrected their reduced Ca^2+^ influx. We conclude that the absence of EPHB6 and the presence of testosterone would lead to augmented large conductance calcium-activated potassium channel currents, which limit voltage-gated calcium channel opening in adrenal gland chromaffin cells. Consequently, acetylcholine-triggered Ca^2+^ influx is reduced, leading to lower catecholamine release in adrenal gland chromaffin cells from male knockout mice. This explains the reduced resting-state blood catecholamine levels, and hence the blood pressure, in male but not female EPHB6 knock mice. These findings have certain clinical implications.

## Introduction

Erythropoietin-producing hepatocellular receptors (EPHs), the largest family of receptor tyrosine kinases, comprise about 25 percent of known receptor tyrosine kinases^1^. They are divided into A and B subfamilies (EPHAs and EPHBs), based on sequence homology. The EPHA subfamily has nine members, and EPHB has six members. Their ligand ephrins (EFNs) are also cell surface molecules^1,2^, which are also classified into A and B subfamilies (EFNAs and EFNBs) based on the way they anchor on the cell surface. EFNAs bind to the cell surface via glycosylphosphatidylinositol, while EFNBs are transmembrane proteins. The signaling from their ligand EFNs to EPHs is called forward signaling. EFNs, although ligands, can also transduce signals into cells^2^, and signaling from EPHs to EFNs is called reverse signaling. Interactions among EPHs and EFNs are promiscuous: a given EPH can interact with multiple EFNs and *vice versa*. In general, EPHA members bind preferentially to EFNA members, as EPHB members do to EFNB members^2^.

EPHs/EFNs function in many organs and systems^2^. Our laboratory was the first to report the critical roles of EPHs and EFNs in the immune system^3–15^. In the past five years, we have discovered novel functions of EPHs/EFNs in regulating blood pressure^16–21^. We reported that while EPHB6, EFNB1 and EFNB3 deletion results in blood pressure elevation, EPHB4 and EFNB2 deletion reduces it. Thus, EPHBs and EFNBs are a novel yin and yang system that finely tunes blood pressure homeostasis. In all such cases, sex hormones act in concert with these EPHs/EFNs for blood pressure regulation. We have established that vascular smooth muscle cells are target tissues for the blood pressure-regulating effect of these molecules. EPHB6, however, also targets cells responsible for catecholamine secretion. Male EPHB6 knockout (KO) mice have reduced blood catecholamine levels in a resting state^16^, which counteracts the outcome of increased vascular smooth muscle cell contractility, resulting in normal blood pressure. Castration of male KO mice leads to blood catecholamines returning to the normal level^16^. This, concomitantly with enhanced vascular smooth muscle cell contractility, results in blood pressure elevation in these castrated KO mice. This body of evidence indicates that EPHB6 and male sex hormones are acting in concert to regulate catecholamine secretion and blood pressure.

In the present study, we investigated the mechanism by which EPHB6 regulates adrenal gland chromaffin cell catecholamine secretion. We found that adrenal gland chromaffin cells from male KO mice were characterized by a reduced acetylcholine-dependent Ca^2+^ influx, involving non-genomic effects of testosterone. We further demonstrated that Ca^2+^ influx decrease was the consequence of enhanced large conductance calcium-activated potassium (BK) currents in these cells.

## Materials and Methods

### EPHB6 gene KO mice

EPHB6 KO mice were generated in our laboratory, as described previously^7^. They were backcrossed to the C57BL/6 background for more than 15 generations. Age- and sex-matched wild type (WT) littermates were used as controls. All experiments involving castrated mice were conducted at least three weeks post-operation.

### Reverse transcription-quantitative polymerase chain reaction (RT-qPCR)

mRNA levels of *EPHBS*, *EFNBS* and *BK* channel subunits were measured by RT-qPCR. Total RNA from the adrenal glands, adrenal gland medullae and spleen was extracted with TRIzol® (Invitrogen, Burlington, Ontario, Canada) and reverse-transcribed with iScript^TM^cDNA Synthesis Kit (Bio-Rad Laboratories (Canada) Ltd., Mississauga, Ontario, Canada). The primers used for PCR are listed in Supplementary Table 1. Conditions for the qPCR reactions were as follows: two minutes at 50 °C, two minutes at 95 °C, followed by 40 cycles of 10 seconds at 94 °C, 20 seconds at 58 °C, and 20 seconds at 72 °C. B-actin mRNA levels were considered as internal controls. qPCR signals between 22 and 30 cycles were analyzed. Samples were tested in triplicate, and the data were expressed as signal ratios of target RNA/β-actin mRNA.

### Primary adrenal gland chromaffin cell culture

Mouse adrenal gland chromaffin cells were isolated, as described by Kolski-Andreaco *et al*.^22^, with modifications. Briefly, we obtained adrenal glands from 8- to 10-week-old mice, and fat and cortex were removed from these glands. Papain (P4762, Sigma-Aldrich, Oakville, Ontario, Canada) was activated with 5 mmol/L L-cysteine. Adrenal gland medullae were digested by activated papain in Hank’s buffer (2 medullae/100 μl Hank’s buffer containing four units of activated papain) at 37 °C for 25 min. They were washed twice with Hank’s buffer and then triturated by pipetting in 300 μl Hank’s buffer until they became feather-like. Cells were pelleted at 3,700 *g* for three minutes and re-suspended in Dulbecco’s modified Eagle’s medium (DMEM) containing 15% (v/v) fetal calf serum (FCS) for culture.

### Epinephrine measurements

Adrenal glands were resected from EPHB6 KO and WT mice, and cut in half to expose the medulla. They were then stimulated with 5 mmol/L acetylcholine chloride (A2661, Sigma-Aldrich) in 300 μl Hank’s buffer at room temperature for one minute. Epinephrine levels in the supernatants were measured with Epinephrine Research ELISA kit (BAE-5100, Rocky Mountain Diagnostics, Colorado Springs, CO, USA), according to the manufacturer’s instructions. Samples were tested in duplicate by ELISA.

### Immunofluorescence microscopy

Adrenal gland chromaffin cells were cultured in 6-well plates with cover glass placed at the bottom of the wells. After one day, the cells were washed once with phosphate-buffered saline (PBS) and fixed with 4% (w/v) paraformaldehyde for 20 minutes. They were then blocked with 10% (v/v) FCS in PBS for 20 minutes and incubated overnight at 4 °C with goat anti-mouse EPHB6 antibody (Ab; 2 µg/ml, R&D Systems, Minneapolis, MN, USA). They were then reacted with Alexa-488-conjugated donkey anti-goat Ab (2 µg/ml, Molecular Probes, Eugene, OR, USA) for two hours at room temperature, and imbedded with ProLong^®^ Gold anti-fade reagent (Molecular Probes). Cell staining was examined with a Zeiss microscope.

### Ca^2+^ influx measurements

Acetylcholine-stimulated *Ca*^*2*+^ influx in adrenal gland chromaffin cells was measured by microfluorescence technique^23^. Briefly, isolated adrenal gland chromaffin cells were incubated for 24 hours in DMEM containing 15% (v/v) FCS. The cells were loaded with Fura-2-AM (5 μmol/L) for 60 minutes at 37 °C. They were rinsed once in warm DMEM containing 15% (v/v) FCS without dye and placed in Hank’s balanced salt solution containing 1.26 mmol/L Ca^2+^ at 37 °C. They were stimulated with acetylcholine (5 mmol/L) at 37 °C and imaged for 120 seconds at a rate of approximately one measurement per two seconds for the two excitation wave lengths (the exposure time of a particular experiment varied slightly) with a Zeiss fluorescence microscope. Excitation wavelengths were recorded alternatively at 340 nm and 380 nm, and emission was registered at 510 nm. Signals from more than 15 randomly selected cells were recorded, and the results expressed as ratios of fluorescence intensity at 510 nm excited by 340 nm versus 380 nm.

### Whole-cell patch clamping

Adrenal gland chromaffin cells were isolated and cultured for 24–48 hours. They were voltage-clamped to measure calcium and potassium current densities at 20–22 °C, with the perforated whole-cell (amphotericin B; 200μg/mL) configuration of the patch clamp technique as described in detail elsewhere^24,25^. For calcium current density monitoring, adrenal gland chromaffin cells were voltage-clamped at −70 mV, and 200-ms depolarizing pulses were applied at 5-mV steps from −50 to +45 mV, to construct I-V curves. For potassium current density measurement, adrenal gland chromaffin cells were voltage-clamped at −70 mV, and 750-ms depolarizing pulses were applied at 10-mV steps from −70 to +100 mV, in the absence or presence of paxilline, a BK channel blocker. Membrane currents were recorded and normalized to cell capacitance (I(pA/pF)) and current density-voltage curves were generated.

### Ethics statement

All animal studies were approved by the Animal Protection Committee (Comité institutionnel d’intégration de la protection des animaux) of the CRCHUM. All experiments were conducted in accordance with relevant guidelines and regulations of the local government.

### Statistical analysis

All results are presented as means ± S.E. The data were analyzed statistically by 2-tailed Student’s *t*-test, or a linear mixed-effect model (with genotype, individual cell, sex, sex hormone and time as qualitative factors). *P-*values of <0.05 are considered to be statistically significant.

## Results

### Reduced epinephrine secretion by adrenal glands from male but not female EPHB6 KO mice

**Figure 1:**
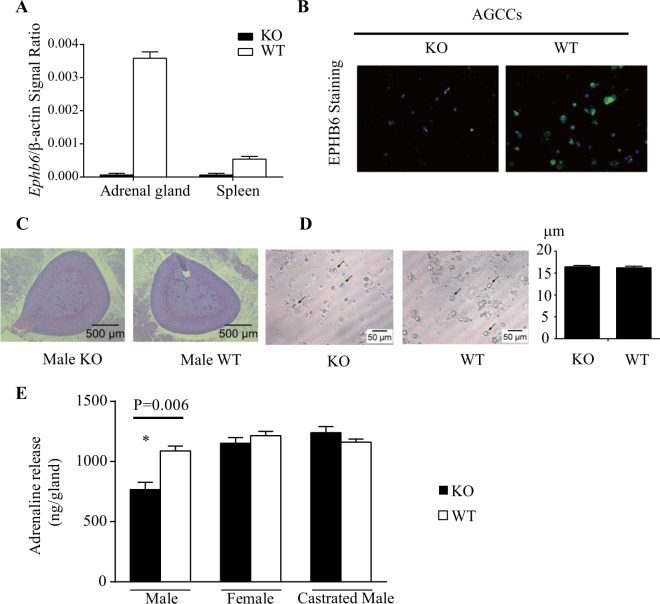
Characterization of adrenal glands and adrenal gland chromaffin cells of EPHB6 KO mice. For A, B and C, experiments were conducted three times and representative data are presented. AGCCs: adrenal gland chromaffin cells. (**A**) EPHB6 mRNA deletion in the adrenal glands and spleen of EPHB6 KO mice. Total RNA was extracted from the adrenal glands and spleen of male WT and EPHB6 KO mice and analyzed by RT-qPCR for EPHB6 mRNA levels. Beta-actin levels were used as internal controls. Samples in RT-qPCR were in triplicate, and EPHB6/β-actin signal ratios are shown as means ± S.E. (**B**) EPHB6 deletion in adrenal gland chromaffin cells from EPHB6 KO mice according to immunofluorescence. Adrenal gland chromaffin cells isolated from adrenal glands of male WT and EPHB6 KO mice were cultured for one day, and then stained with goat anti-mouse EPHB6 Ab followed by Alexa-488-conjugated donkey anti-goat Ab (green). Nuclei were stained with DAPI (blue). (**C**) Normal histology of EPHB6 KO adrenal glands. Sections of adrenal glands from 8- to 10-week-old male WT and EPHB6 KO mice were stained with hematoxylin/eosin. (**D**) Adrenal gland chromaffin cells from male WT and EPHB6 KO and WT mice are similar in size. Left panel: phase-contrast micrographs of adrenal gland chromaffin cells from WT and KO mice after 24-hour culture. Right panel: diameters of adrenal gland chromaffin cells from WT and KO mice after 24-hour culture. Means ± S.E. of the diameters of more than 30 adrenal gland chromaffin cells (more than 10 cells/mouse and three mice/group) from male WT and KO mice are shown. No significant difference is observed (2-way Student’s *t* test). (**E**) Epinephrine release from the adrenal glands of WT and KO mice. Adrenal glands, isolated from male, female and castrated male KO and WT mice, were cut in half, and were stimulated with acetylcholine (5 mmol/L) in 300 μl Hank’s buffer for one minute at room temperature. The supernatants were analyzed for epinephrine levels by ELISA. Data from three independent experiments (each using one KO mouse and one WT control) were pooled, analyzed by two-way Student’s *t* test, and reported as means ± S.E. *p < 0.05.

**Figure 2:**
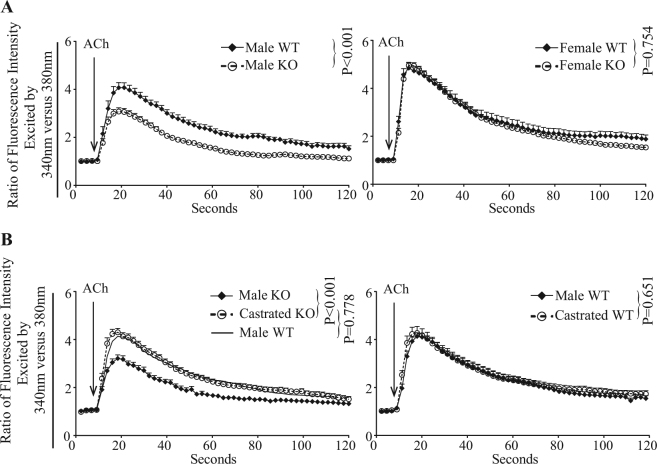
Ca^2+^ influx in adrenal gland chromaffin cells from EPHB6 KO and WT mice. Adrenal gland chromaffin cells were loaded with Fura2-AM, and stimulated with acetylcholine (5 mmol/L) at 37 °C. The cells were imaged for 120 seconds at a rate of one measurement per two seconds. Arrows indicate the time points where acetylcholine was added. Signals from more than 15 randomly selected cells per group were recorded, and the results expressed as means ± S.E. of ratios of fluorescence intensity at 510 nm excited by 340 nm versus 380 nm. The data were analyzed in a linear mixed-effect model, with genotype, individual cells, sex and time as qualitative factors. *P*-values are indicated. The experiments were repeated at least three times. Data from representative experiments are shown. (**A**) Male but not female KO adrenal gland chromaffin cells present reduced Ca^2+^ influx compared to their WT counterparts. (**B**) Castration reverses low Ca^2+^ influx in KO adrenal gland chromaffin cells to normal but has no effect on WT adrenal gland chromaffin cells. The S.E. of the solid line curve representing male WT adrenal gland chromaffin cell Ca^2+^-influx in the left panel is omitted for better visualization.

We previously reported that male EPHB6 KO mice present decreased 24-hour urine catecholamine levels^16^. To establish causality of this phenotype to the adrenal glands, we measured the KO adrenal gland catecholamine secretion by RT-PCR. As anticipated, RT-qPCR showed that the adrenal gland from KO mice did not express EPHB6 at the mRNA level (Fig. 1A). No compensatory mRNA up-regulation of other EPHB members, such as EPHB1, EPHB2, EPHB3 or EPHB4, was evident in adrenal glands (Supplementary Figure 1 (S. Figure 1) from male, female or castrated KO mice. Nor was there abnormal expression of mRNA levels of EPHB6 ligands (EFNB1, EFNB2 and EFNB3) in these KO adrenal gland chromaffin cells (S. Figure 2). EPHB6 protein expression was undetectable by immunofluorescence in KO adrenal gland chromaffin cells (Fig. 1B). KO adrenal glands were of similar size as WT counterparts and showed no histological abnormalities (Fig. 1C). No significant difference in the size of adrenal gland chromaffin cells isolated from KO versus WT adrenal glands was observed (Fig. 1D). Acetylcholine-stimulated catecholamine secretion by KO adrenal glands was then assessed with epinephrine as a representative catecholamine. Adrenal glands from male but not female KO mice presented reduced epinephrine secretion, but castration reversed this KO phenotype by augmenting epinephrine secretion to normal levels (Fig. 1E). This observation is consistent with catecholamine levels in the *in vivo* KO mice, and supports the hypothesis that EPHB6 and male sex hormones jointly regulate catecholamine secretion from adrenal gland chromaffin cells.

### EPHB6 and testosterone jointly regulate adrenal gland chromaffin cell Ca^2+^ influx

**Figure 3:**
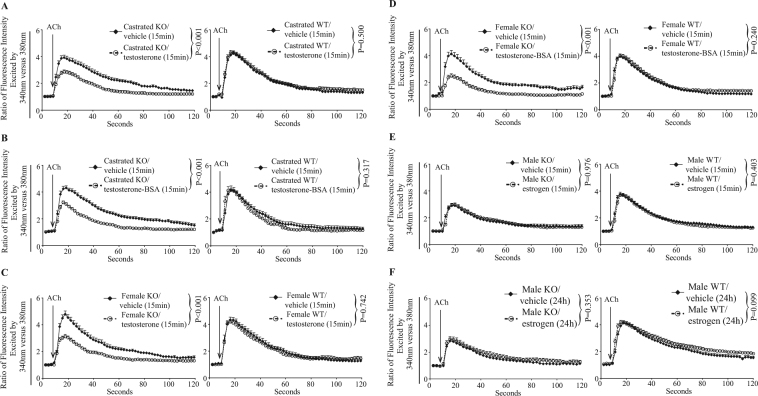
Effects of sex hormones on Ca^2+^ flux in adrenal gland chromaffin cells from EPHB6 KO and WT mice. The experiment procedures and data presentation are the same as described in Fig. 2. The data were analyzed in a linear mixed-effect model, with genotype, individual cells, sex, sex hormone and time as qualitative factors. (**A**) Fifteen-minute testosterone treatment rapidly lowers Ca^2+^ influx in castrated KO but not in WT adrenal gland chromaffin cells. (**B**) Cell membrane-impermeable, BSA-conjugated testosterone rapidly (within 15 minutes) lowers Ca^2+^ influx in castrated KO but not WT adrenal gland chromaffin cells. (**C**) Fifteen-minute testosterone treatment rapidly lowers Ca^2+^ influx in female KO but not WT adrenal gland chromaffin cells. (**D**) Cell membrane-impermeable, BSA-conjugated testosterone rapidly (within 15 minutes) lowers Ca^2+^ influx in female KO but not WT adrenal gland chromaffin cells. (**E**) Short-term (15-minute) estrogen treatment does not affect male KO and WT adrenal gland chromaffin cell Ca^2+^ influx. F. Long-term (24-hour) estrogen treatment does not affect male KO and WT adrenal gland chromaffin cell Ca^2+^ influx.

Ca^2+^ influx is the main trigger for catecholamine release in adrenal gland chromaffin cells. Defective catecholamine release in adrenal gland chromaffin cells from male KO mice prompted the examination of Ca^2+^ influx in these cells. Acetylcholine-stimulated Ca^2+^ influx was significantly reduced in adrenal gland chromaffin cells from male but not female KO mice (Fig. 2A), corroborating the catecholamine phenotype *in vivo*. Castration rescued Ca^2+^ influx in KO adrenal gland chromaffin cells to a level similar to that of their WT counterparts, while it had no impact on that of adrenal gland chromaffin cells from WT males (Fig. 2B). To disentangle the effects between sex hormones and sex, we treated adrenal gland chromaffin cells from castrated males with testosterone. While Ca^2+^ influx in adrenal gland chromaffin cells from castrated WT mice was not affected by 15-minute testosterone treatment, it was augmented in adrenal gland chromaffin cells from castrated KO mice to the level of their WT counterparts (Fig. 3A), indicating that testosterone rather than sex influenced Ca^2+^ influx. The rapid response of adrenal gland chromaffin cells to testosterone treatment (15 minutes) also suggests that the effect is non-genomic. To confirm this finding, adrenal gland chromaffin cells were treated with bovine serum albumin (BSA)-conjugated testosterone, which cannot penetrate the cell membrane and can only exert non-genomic effects. Again, similarly to regular testosterone, 15-minute treatment with this membrane-impermeable testosterone augmented Ca^2+^ influx in adrenal gland chromaffin cells from castrated KO mice, but not in adrenal gland chromaffin cells from castrated WT mice (Fig. 3B), indicating that the effect is indeed non-genomic.

Based on these findings, testosterone-induced Ca^2+^ influx suppression in the absence of EPHB6 is expected in adrenal gland chromaffin cells from female KO mice. Indeed, 15-minute testosterone treatment reduced Ca^2+^ influx in adrenal gland chromaffin cells from female KO but not in WT mice (Fig. 3C). Moreover, BSA-conjugated cell membrane-impermeable testosterone was equally effective in these adrenal gland chromaffin cells from female KO mice (Fig. 3D), supporting non-genomic effects of the testosterone on adrenal gland chromaffin cells Ca^2+^ influx.

The lack of estrogen as a potential cause for the observed reduced Ca^2+^ influx in adrenal gland chromaffin cells from male KO mice was then explored. Adrenal gland chromaffin cells from male KO mice were exposed to estrogen for 15 minutes (Fig. 3E) or 24 hours (Fig. 3F). Regardless of the exposure time, estrogen had no effect on adrenal gland chromaffin cells from male KO or WT mice in terms of acetylcholine-stimulated Ca^2+^ influx. This strongly suggests that testosterone alone, but not estrogen, in concert with EPHB6, regulates adrenal gland chromaffin cell Ca^2+^ influx.

### Effects of EPHB6 and male sex hormone on adrenal gland chromaffin cell electrophysiological properties

**Figure 4:**
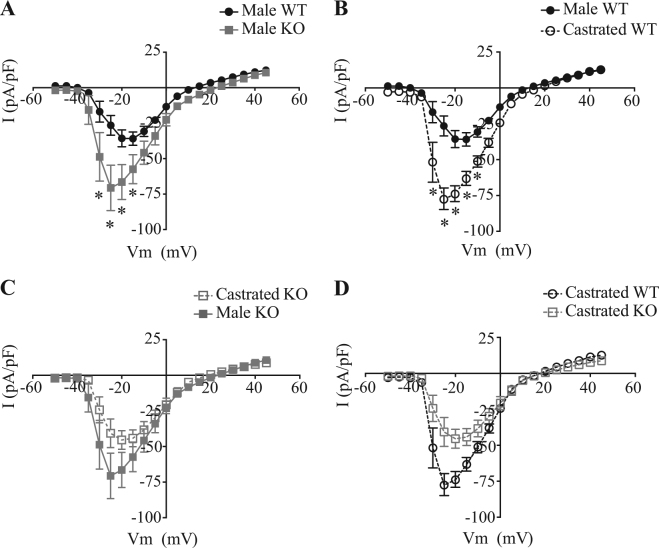
Voltage-dependent calcium current densities in adrenal gland chromaffin cells from WT and EPHB6 KO mice, with or without castration. Total calcium current densities recorded in adrenal gland chromaffin cells with the perforated patch technique. Current-voltage curves are presented as means ± S.E. of I(pA/pF). Curves are generated from pooled data from 8–10 cells from 2–3 mice: WT (n = 11 cells from 3 mice), KO (n = 8 cells from 2 mice), castrated WT (n = 10 cells from 3 mice), castrated KO (n = 8 cells from 3 mice). *p < 0.05 (2-tailed Student’s *t* test). (**A**) Male KO adrenal gland chromaffin cells showed higher Ca^2+^current densities compared to their WT counterparts. (**B**) Castration significantly increased voltage-gated Ca^2+^ current densities in WT adrenal gland chromaffin cells. (**C**) Castration did not significantly increase voltage-gated Ca^2+^ current densities in KO adrenal gland chromaffin cells. (**D**) Voltage-gated Ca^2+^ current densities became similar in adrenal gland chromaffin cells from castrated WT and KO adrenal gland chromaffin cells.

Voltage-gated calcium channel function in adrenal gland chromaffin cells from KO mice was electrophysiologically assessed using the perforated patch clamp technique (Fig. 4). According to the calcium influx data, smaller calcium current densities were expected. Unexpectedly, calcium current densities were significantly higher in male KO adrenal gland chromaffin cells (Fig. 4A). Whilst castration increased Ca^2+^ current densities in WT adrenal gland chromaffin cells (Fig. 4B), no significant changes were observed in male KO adrenal gland chromaffin cells (Fig. 4C). As a consequence, castration abolished the difference in calcium current-voltage relationships between WT and KO adrenal gland chromaffin cells (Fig. 4D). It should be noted that although the deletion of EPHB6 and the presence of testosterone could potentially increase voltage-gated calcium channels current densities during patch clamping, such increase might not happen in an intact cell under a physiological condition (otherwise, the Ca^2+^ influx would have increased in male KO adrenal gland chromaffin cells), and hence the alternation of voltage-gated calcium channel function is not the direct cause for the EPHB6-associated decrease in Ca^2+^ influx of male KO adrenal gland chromaffin cells.

**Figure 5:**
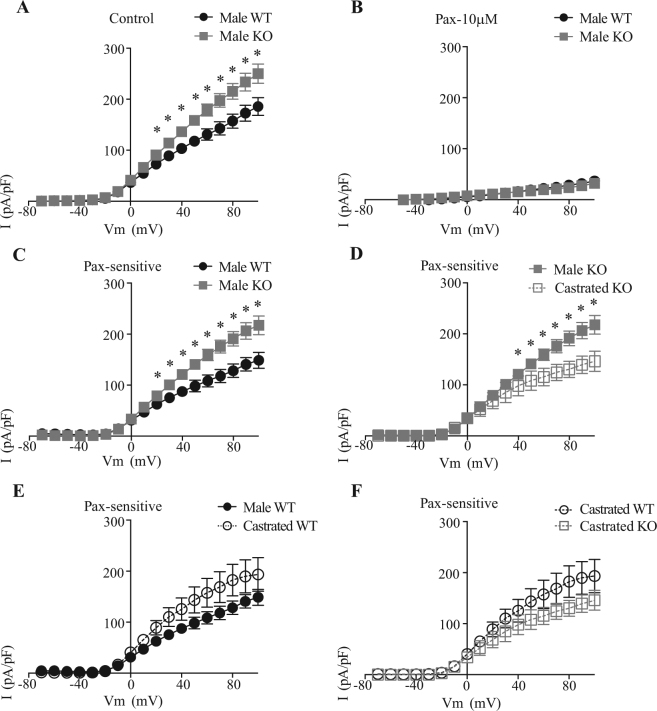
BK channel current densities in adrenal gland chromaffin cells from WT and KO mice, with or without castration. Outward potassium current densities were recorded with the perforated patch clamp technique in the absence or presence of paxilline (Pax; 10 μM), a BK channel blocker. Current-voltage curves are presented as means ± S.E. of I(pA/pF). Curves are generated from pooled data from 4–12 cells from 2–5 different mice: WT (n = 7 cells from 4 mice), KO (n = 12 cells from 3 mice), castrated WT (n = 8 cells from 5 mice), castrated KO (n = 4 cells from 2 mice). *p < 0.05 (2-tailed Student’s *t* test). (**A**) KO adrenal gland chromaffin cells showed higher voltage-gated K^+^ current densities. (**B**) Voltage-gated K^+^ current densities were almost abolished by the BK channel blocker paxilline. (**C**) KO adrenal gland chromaffin cells showed higher voltage-gated, paxilline-sensitive K^+^ current densities. (**D**) Castration significantly decrease voltage-gated paxilline-sensitive K^+^ current densities in KO adrenal gland chromaffin cells. (**E**) Castration did not significantly change voltage-gated, paxilline-sensitive K^+^ current densities in WT adrenal gland chromaffin cells. (**F**) Paxilline-sensitive BK current densities from WT and KO adrenal gland chromaffin cells become similar after castration.

Indirect modulation of voltage-gated calcium channels activity, and subsequent calcium influx, can occur through the regulation of membrane potential. Opening of BK channels strongly hyperpolarizes cell membrane and thus leads to the closure of voltage-gated calcium channels in adrenal gland chromaffin cells. The impact of EPHB6 and testosterone on BK channel function was investigated. Outward K^+^ currents were significantly larger in adrenal gland chromaffin cells from male KO mice compared to their WT counterparts (Fig. 5A). Paxilline, a BK channel blocker, almost completely abolished the currents in both groups (Fig. 5B). This indicates that the outward K^+^ currents in WT and KO adrenal gland chromaffin cells occur mainly via BK channels. The paxilline-sensitive BK channel currents were significantly higher in male KO adrenal gland chromaffin cells than that in WT adrenal gland chromaffin cells (Fig. 5C), and castration significantly decreased BK currents in KO but not WT adrenal gland chromaffin cells (Fig. 5D and E). Therefore, BK currents became similar in WT and KO adrenal gland chromaffin cells after castration (Fig. 5F).

We identified BK function as a critical link in response to testosterone with regard to Ca^2+^ influx in the EPHB6 KO adrenal gland chromaffin cells. *In vitro* (or rather, *ex vivo*), the Ca^2+^ current density of adrenal gland chromaffin cells of the KO mice was rapidly augmented within 15 minutes by testosterone, suggesting non-genomic effect of the androgen. However, this cannot exclude the possibility that *in vivo*, testosterone and EPHB6 also affect BK channel expression through genomic effects via classical nuclear androgen receptors^26^, which would increase BK current densities *in vivo* or *ex vivo*. Besides, *in vivo*, the longer time frame will allow the rapid non-genomic signaling of testosterone via Src kinases^27^ or cell surface androgen receptors^28^ to travel to the nuclei, converting non-genomic effects to genomic effects. Therefore, it is necessary to evaluate the BK expression *ex vivo* from WT and KO chromaffin cells. BK channels are composed of four pore-forming alpha subunits and a regulatory beta-subunit isoform^29^. We probed for their expression in mouse adrenal gland chromaffin cells, and found that the beta 2 subunit (KCNMB2) was the dominant beta subunit isoform in these cells (S. Figure 3). However, no difference in the expression of either the alpha or beta subunit mRNA was detected in adrenal gland chromaffin cells from KO males WT in males or from castrated KO and WT males. This suggests that the regulation of BK channels by EPHB6 and testosterone do not occur at the expression level.

**Figure 6:**
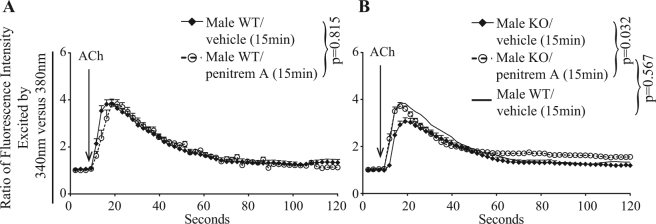
The BK channel blocker penitrem A augments acetylcholine-triggered Ca^2+^influx in KO adrenal gland chromaffin cells to a normal level. WT (**A**) and KO (**B**) adrenal gland chromaffin cells from male mice were loaded with Fura2-AM for one hour, and then treated with a BK channel blocker penitrem A (10 μmol/L) for 15 minutes. The cells were then washed and stimulated with acetylcholine at 37 °C. Their Ca^2+^ flux was recorded for 120 seconds at a rate of measurement per two seconds. Arrows indicate time points where acetylcholine was added. Signals from more than 15 or more randomly-selected cells per group were recorded, and the results expressed as means ± S.E. of ratios of fluorescence intensity at 510 nm excited by 340 nm versus 380 nm. In B, the means of WT adrenal gland chromaffin cell signals were presented as a thin line without S.E. for better viewing. The data were analyzed by linear mixed-effect model, with genotype, individual cells, penitrem A and time as qualitative factors. *P*-values are indicated. Experiments were conducted at least three times. Data from representative ones are shown.

Our data indicate that enhanced BK currents in male KO adrenal gland chromaffin cells might lead to decreased open probability of voltage-gated calcium channels, reducing Ca^2+^ influx in these cells. If so, blocking BK channels would prevent voltage-gated calcium channel inhibition and increase Ca^2+^ influx in male KO adrenal gland chromaffin cells. To test this hypothesis, adrenal gland chromaffin cells were treated with a BK channel inhibitor, penitrem A. Ca^2+^ influx was unaltered by penitrem A in male WT adrenal gland chromaffin cells (Fig. 6A), but it was significantly increased in KO adrenal gland chromaffin cells, reverting to WT control levels (Fig. 6B).

## Discussion

The present study revealed that EPHB6 deletion decreased male but not female adrenal gland chromaffin cell Ca^2+^ influx and catecholamine secretion. Testosterone was required for this phenotype, as the absence of both EPHB6 and testosterone reversed the KO adrenal gland chromaffin cell phenotype to that of their WT controls. The functional mechanism involves BK channels.

**Figure 7:**
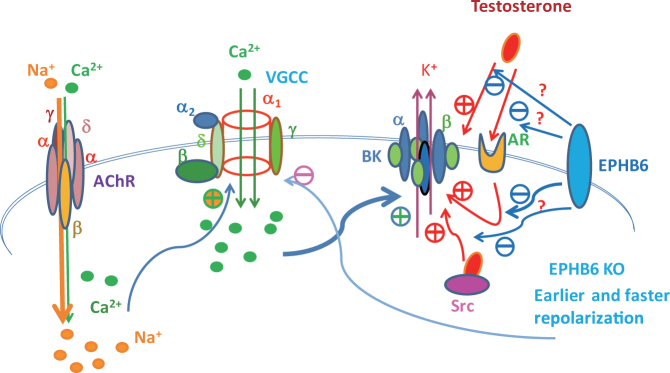
A model illustrating the concerted effect of EPHB6 and testosterone in regulating Ca^2+^ influx in adrenal gland chromaffin cells. In adrenal gland chromaffin cells, acetylcholine (ACh) stimulation of acetylcholine receptors (AChR) causes initial depolarization by allowing a small amount of Ca^2+^ influx plus a large amount of Na^+^ influx. The combined effect of both inward cation fluxes opens voltage-gated calcium channels for larger Ca^2+^ influx. BK channels are then activated by depolarization as well as by increased Ca^2+^ concentration, allowing K^+^ ions efflux, therein repolarizing the cells. As a consequence, voltage-gated calcium channels (VGCC) are shut down, terminating Ca^2+^ influx. Testosterone promotes the BK currents by direct binding with the BK channel, or by non-genomic signaling via its cell surface androgen receptors (AR) or via intracellular Src kinases. EPHB6 might interfere with the testosterone-BK association, or block the interaction between testosterone and AR, or suppress Src signaling. These 3 possible mechanisms are to be tested, and hence marked with question marks. As a consequence, the presence of EPHB6 suppresses the positive effect of testosterone on the BK currents. EPHB6 KO liberates this positive impact, leading to a larger K^+^ outflow. This results in earlier and faster repolarization, an earlier closure of voltage-gated calcium channels, and the subsequently reduced Ca^2+^ influx.

Ca^2+^ influx is an essential signal triggering catecholamine release from adrenal gland chromaffin cells. Our data shows that this Ca^2+^ influx is compromised in male EPHB6 KO adrenal gland chromaffin cells. The electrophysiology of adrenal gland chromaffin cells related to Ca^2+^ influx is depicted in Fig. 7. In adrenal gland chromaffin cells, acetylcholine stimulation of acetylcholine receptors evokes a small Ca^2+^ influx plus a large Na^+^ influx. The combined effect of these inward cations causes depolarization, which increases voltage-gated calcium channels open probability and leads to a larger Ca^2+^ influx^30^. Ensuing BK channel activation re-hyperpolarizes adrenal gland chromaffin cell membrane^31^. This re-hyperpolarization reduces voltage-gated calcium channels’ opening and thus terminates Ca^2+^ influx^32^. The decreased calcium influx and catecholamine release observed in male EPHB6 KO mice could have resulted from a decrease in calcium channels’ function. However, perforated patch recordings show voltage-gated calcium channels’ current densities were rather larger in adrenal gland chromaffin cells from KO compared to that of their WT littermates. These results strongly suggest that EPHB6 modulation of calcium influx is not due to direct suppression of voltage-gated calcium channel function.

Alternatively, EPHB6’s control of Ca^2+^ influx could be indirect through stabilizing (or repolarizing) the membrane potential of adrenal gland chromaffin cells. Given the high input resistance of adrenal gland chromaffin cells, a small modification of membrane conductance would be sufficient to abolish or substantially limit membrane depolarization. K^+^ currents thus play a major role in controlling membrane potential of adrenal gland chromaffin cells and catecholamine release. Indeed, K^+^ currents appear to be modified in male KO adrenal gland chromaffin cells. BK channels were the main K^+^ conductance altered by EPHB6 expression, as their current densities were in accordance with the adrenal gland chromaffin cell phenotype observed. In male KO adrenal gland chromaffin cells, BK current densities were substantially increased (Fig. 5C), and castration abolished such increase (Fig. 5D and F). We thus propose the model illustrated in Fig. 7 to portray the mechanism by which EPHB6 and testosterone act in concert to modulate adrenal gland chromaffin cell Ca^2+^ influx. Testosterone enhances BK currents either through direct binding with the ion channel^33^, by non-genomic signaling through Src kinases^27^ or its cell surface androgen receptors^28^. On the other hand, EPHB6 might interfere with the testosterone-BK channel, testosterone-androgen receptor, or testosterone-Src interactions (to be confirmed, and hence marked with question marks). As a consequence, the presence of EPHB6 suppresses the positive effect of testosterone on BK channel activity. Abolished expression of EPHB6 in KO mice alleviates the inhibitory influence of testosterone over BK channels, leading to a larger K^+^ efflux. This evokes an earlier and faster repolarization, decreases sustained voltage-gated calcium channels’ probability to open, and subsequently reduces Ca^2+^ influx.

This proposed model (Fig. 7) is validated by our experimental data. Indeed, a BK channel blocker, penitrem A, effectively augmented Ca^2+^ influx in male KO adrenal gland chromaffin cells, corroborating the critical role of BK channel activity in decreasing Ca^2+^ influx in these cells. The current literature also supports our model. Testosterone has been shown to increase the BK current^34^. A recent report indicated that testosterone can interact directly with BK channel subunits in rat anterior pituitary tumor cells and modulate their function in patch-clamp assays^33^. Therefore, direct testosterone binding to BK channels in adrenal gland chromaffin cells might enhance BK channel activity. BK channel function would not be altered in male WT adrenal gland chromaffin cells by testosterone, because EPHB6 blocks BK channel’s association with the latter. BK currents are not altered in female KO adrenal gland chromaffin cells either, despite the absence of EPHB6, because of low testosterone levels. Moreover, BK channel currents are normal in female WT adrenal gland chromaffin cells, due to both the absence of testosterone and the presence of EPHB6. To test these hypotheses, we conducted immunoprecipitation and fluorescence resonance energy transfer to assess physical interactions between EPHB6 and KCNMB2, the major regulatory β subunit, but to no avail (data not shown). Limitations of the approaches, such as assay sensitivity and affinity between EPHB6 and BK β-subunit, could account for such negative results. Further investigation in this regard would thus be required.

Androgens have two major forms: testosterone and 5α-dihydrotestosterone. The latter is a metabolite of the former, and binds with higher affinity to androgen receptors^35^. Classical androgen receptors are intracellular proteins, and upon binding to testosterone or 5α-dihydrotestosterone, they translocate into nuclei and serve as DNA-binding transcription factors that regulate genes with androgen-responsive elements^36^. This is called genomic action. Androgen receptors can also interact directly with Src kinases and trigger their activation^27^. Cell membrane androgen receptors exist too, and they may be G protein–associated but are poorly characterized^28,37–39^. The latter two actions are non-genomic.

Our study *in vitro* reveals that the non-genomic effect of testosterone appears to be sufficient to cause increased Ca^2+^ influx in male KO adrenal gland chromaffin cells. It is likely that this observed androgen effect is not unique for testosterone; other testosterone derivatives, such as the more potent 5α-dihydrotestosterone, might also be effective. This will need to be confirmed. Such *in vitro* non-genomic effect does not exclude the existence of possible *in vivo* genomic effect of androgens, as they are not mutually exclusive. The possible *in vivo* genomic effect of androgen on Ca^2+^ influx could be confirmed or refuted by using mice with adrenal gland-specific null mutation of the classical intracellular androgen receptor. To the best of our knowledge, such a study has not been conducted and would be interesting to perform.

It is conceivable that males with EPHB6 loss-of-function mutations might become hypertensive later in life because of decreased testosterone levels, which may cause them to lose the blood pressure-lowering beneficial effect of EPHB6 mutation by increasing their resting-state catecholamine levels. Consequently, their blood pressure might increase due to other genetic and environmental factors, as hypertension is a polygenic and multifactorial disease. For these patients, testosterone replacement therapy might restore the protective influence of EPHB6 mutation in lowering catecholamine secretion and hence, blood pressure.

The effect of testosterone on blood pressure is often controversial. In animal studies, castration often results in lower blood pressure^40–42^. In humans, different studies reported mixed results, probably due to the heterogeneity of the human population. Male hypogonadism is known to associate with hypertension^43–48^. There are multiple articles showing blood pressure reduction after testosterone replacement therapy^49–52^. These and additional favorable results with regard to testosterone replacement therapy in reducing cardiovascular risks including blood pressure are discussed in two recent review papers^53,54^. It is true that a couple of large retrospective studies have revealed that testosterone replacement therapy is associated with increased cardiovascular risks^55,56^. However, the conclusions from these studies are disputed as “retrospective, highly statistical and only with a minor effect size” and “are unlikely reproducible or accurate”^54^. It is safe to say that the effects of testosterone replacement therapy on cardiovascular risks are not conclusive.

The results of clinical studies are rarely black and white (*i.e*., with 100% penetrance). Rather, the conclusions depend heavily on statistical analysis, which has the limitation of discounting the responses of a subpopulation within the case group. For example, in a drug efficacy study, if we assume a placebo effect occurs in 20% of a control group, and a positive therapeutic effect of 35% in a drug-treated group, which represents a very moderate efficacy, we will only need 138 individuals each in the control and treated group to achieve 80% power to arrive at a conclusion that the treatment is statistically effective. In this case, the fate of 65% of treated but non-responsive patients is practically ignored. In a real example of using diuretics in treating acute congestive heart failure, more than 50% of the treated patients are not responding to 40 mg/furosemide in terms of body weight reduction^57^, and yet furosemide is still used to treat this condition. For the same reason but in a reverse way, the statistically significant side-effect of testosterone replacement therapy does not mean that the side-effect occurs in all the individuals. It is totally possible that for a subpopulation of hypogonadic patients with EPHB6 loss-of-function mutations, testosterone replacement therapy does not cause increased cardiovascular risks, but reduces resting state catecholamine secretion, and hence their blood pressure. For these individuals, testosterone might be a causative rather than symptomatic treatment for their hypertension. After all, testosterone administered in a proper dosage and format will restore what they used to have in sufficient quantity in their adult life.

The potential risk of testosterone in promoting prostate cancer is a concern. However, this is mitigated because patients with prostate cancer or elevated prostates-specific antigen levels are routinely excluded from testosterone replacement therapy. Also, multiple short- and long-term (five years) testosterone replacement therapy studies have found no evidence of prostate-specific antigen level increases^49,52,58^, further alleviating the concern.

The current anti-hypertensive drugs are highly effective with minimal side-effects. This has made the entry threshold of testosterone as a new therapeutic agent much higher. However, in the age of personalized medicine, for those whose hypertension is caused by EPHB6 mutations and subsequent hypogonadism, if testosterone could address the cause rather than symptoms, its therapeutic application should be considered.

The present study reveals a novel mechanism by which EPHB6 and testosterone jointly regulate catecholamine release from adrenal gland chromaffin cells. The results might explain some controversial findings with regard to cardiovascular benefits or risks after testosterone replacement therapy. Further, this study has raised a possibility of using testosterone as a personalized medicine to lower resting-state catecholamine secretion and hence, blood pressure.

## Electronic supplementary material

Supplementary data
