# Supplementary material for: EPHB6 and testosterone in concert regulate epinephrine release by adrenal gland chromaffin cells
Source: Sci Rep. 2018 Jan 16;8:842. doi: 10.1038/s41598-018-19215-2 (PMC5770418; doi:10.1038/s41598-018-19215-2)
Supplement: Supplementary file 1 — Supplementary data [file 41598_2018_19215_MOESM1_ESM.doc]

**EPHB6 and testosterone in concert regulate epinephrine release by adrenal gland chromaffin cells**

^a,d*^Yujia Wang, ^a*^Wei Shi, ^b^Alexandre Blanchette, ^a^Junzheng Peng, ^a^Shijie Qi, ^a^Hongyu Luo, ^b^Jonathan Ledoux, and ^a,c^Jiangping Wu

**Running title**: *EPHB6 and catecholamine release*

^a^Research Centre, Centre hospitalier de l’Université de Montréal (CRCHUM), Montreal, Quebec,Canada H2X 0A9; ^b^Montreal Heart Institute, Montreal, Quebec, Canada H1T 1C8; ^c^Nephrology Department, CHUM, Montreal, Quebec, Canada H2L 4M1; ^d^The Children’s Hospital, Zhejiang University School of Medicine, Hangzhou, Zhejiang, China 310003.

**SUPPLEMENTARY TABLE**

**Supplementary Table 1.RT-qPCR primer sequences**

| RT-qPCR primer sequences | | |
| --- | --- | --- |
| Gene | sense sequences | antisense sequences |
|  *β-actin* | 5’-TCGTACCACAGGCATTGTGATGGA-3’ | 5’-TGATGTCACGCACGATTTCCCTCT-3’ |
| *Ephb1* | 5’-ACCATGAGGAGCATCACCTTGTCA-3’ | 5’-TAGCCCATCGATACGTGCTGTGTT-3’ |
| *Ephb2* | 5’-CCAGTGATGTGTGGAGCTATG-3’ | 5’-GGAGGTAGTCTGTAGTCCTGTT-3’ |
| *Ephb3* | 5’-AGTTCGCCAAGGAGATCGATGTGT-3’ | 5’-TCAGCGTCTTGATAGCCACGAACA-3’ |
| *Ephb4* | 5’-CTACGTCTCTAACCTCCCATCT-3’ | 5’-GCTGGTCACCCTTTCTCTTT-3’ |
| *Ephb6* | 5’-CTTTGCCTTTGTTCACCGAGCACT-3’ | 5’-AGCAAGGAACTTGAACCCTGAGGA-3’ |
| *Efnb1* | 5’-ACCAGGAAATCCGCTTCACCATCA-3’ | 5’-ACAGCATTTGGATCTTGCCCAACC-3’ |
| *Efnb2* | 5’-TTCTGCTGGATCAGCCAGGAATCA-3’ | 5’-TCCTGATGCGATCCCTGCGAATAA-3’ |
| *Efnb3* | 5’-AGTTCCGATCCCACCACGATTACT-3 | 5’-AGAAGCACCTTCATGCCTCTGGTT-3 |
| *Kcnma1* | 5’-CAACGTGTTCTTCCTCCTCTAC-3’ | 5’-CAGGAGGGACTGTGAAGAAATC-3’ |
| *Kcnmb1* | 5’-CCCTGACTTCAGTTGGTTCATA-3’ | 5’-AGAGAGAGCACGTAGGGATAG-3’ |
| *Kenmb2* | 5’-CCAGGTCTCTGTTCTGAGTTTC-3’ | 5’-CACTTGCTACAGGGCTCAATA-3’ |
| *Kcnmb3* | 5’-GGAGTTGTTCTGAGGAAGTCTG-3’ | 5’-GCCTCCCAGCAAAGTCAATA-3’ |
| *Kcnmb4* | 5’-TCCTATATCCCGCCCTGTAA-3’ | 5’-CTGGGAACCGATCTCATCTTT-3’ |
| *Cacna1g* | 5’-GGGAGCAGGAGTATTTCAGTAG-3’ | 5’-GATGTTTCTGCCTGGGTATCT-3’ |
| *Cacna1h* | 5’-TCCTTCTGCTGTGCTTCTTC-3’ | 5’-CAGGAAGGTCAGGTTGTTGT-3’ |
| *Cacna1i* | 5’-GCCCTACTATGCCACCTATTG-3’ | 5’-AGGCAGATGATGAAGGTGATG-3’ |

**Supplementary figures and figure legends**

*
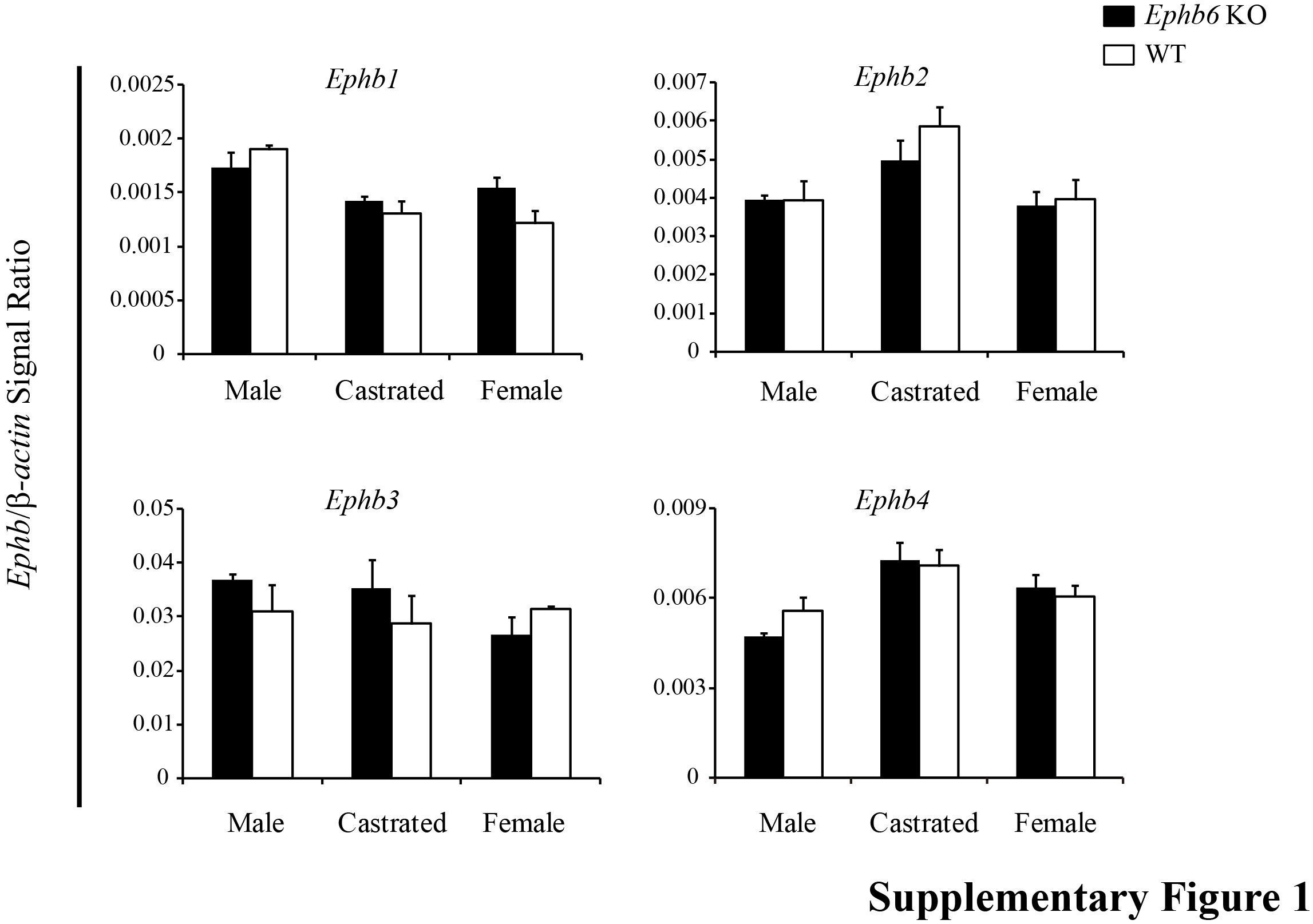
*

*Supplementary Figure 1. Normal expression of Ephb1, Ephb2, Ephb3 and Ephb4 mRNA in adrenal gland medullae from male, female and castrated male WT and EPHB6 KO mice*

Total RNA was extracted from adrenal gland medullae of male and female WT and *Ephb6* KO mice and from castrated WT and KO mice. *Ephb1*, *Ephb2*, *Ephb3* and *Ephb4* mRNA levels were analyzed by RT-qPCR. β-actin levels were used as internal controls. RT-qPCR samples were in triplicate. The experiments were conducted three times, and means ± SEM of *Ephb*/*β-actin* signal ratios of pooled data from the three experiments are shown. Student’s *t* tests were used for statistical analysis, and no significant difference was found among different test groups.

*
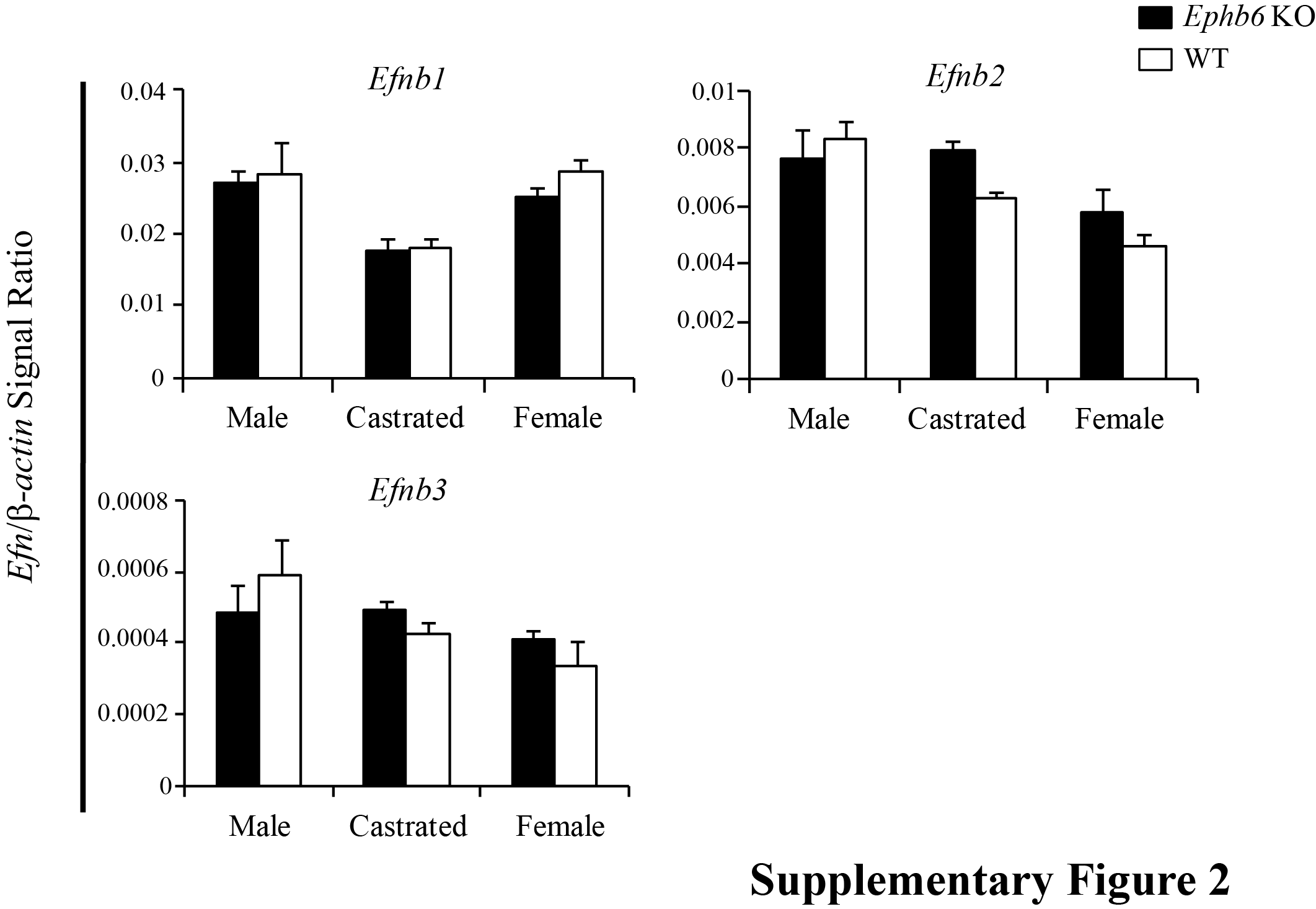
*

*Supplementary Figure 2. Normal Efnb1, Efnb2 and Efnb3mRNA expression in adrenal gland medullae from male, female and castrated male EPHB6 KO mice*

Total RNA was extracted from adrenal gland medullae of male, female and castrated male WT and *Ephb6* KO mice. *Efnb1, Efnb2 and Efnb3* mRNA levels the medullae were measured by RT-qPCR. Experiments and data analysis were conducted as described in Supplementary Figure 1.

*
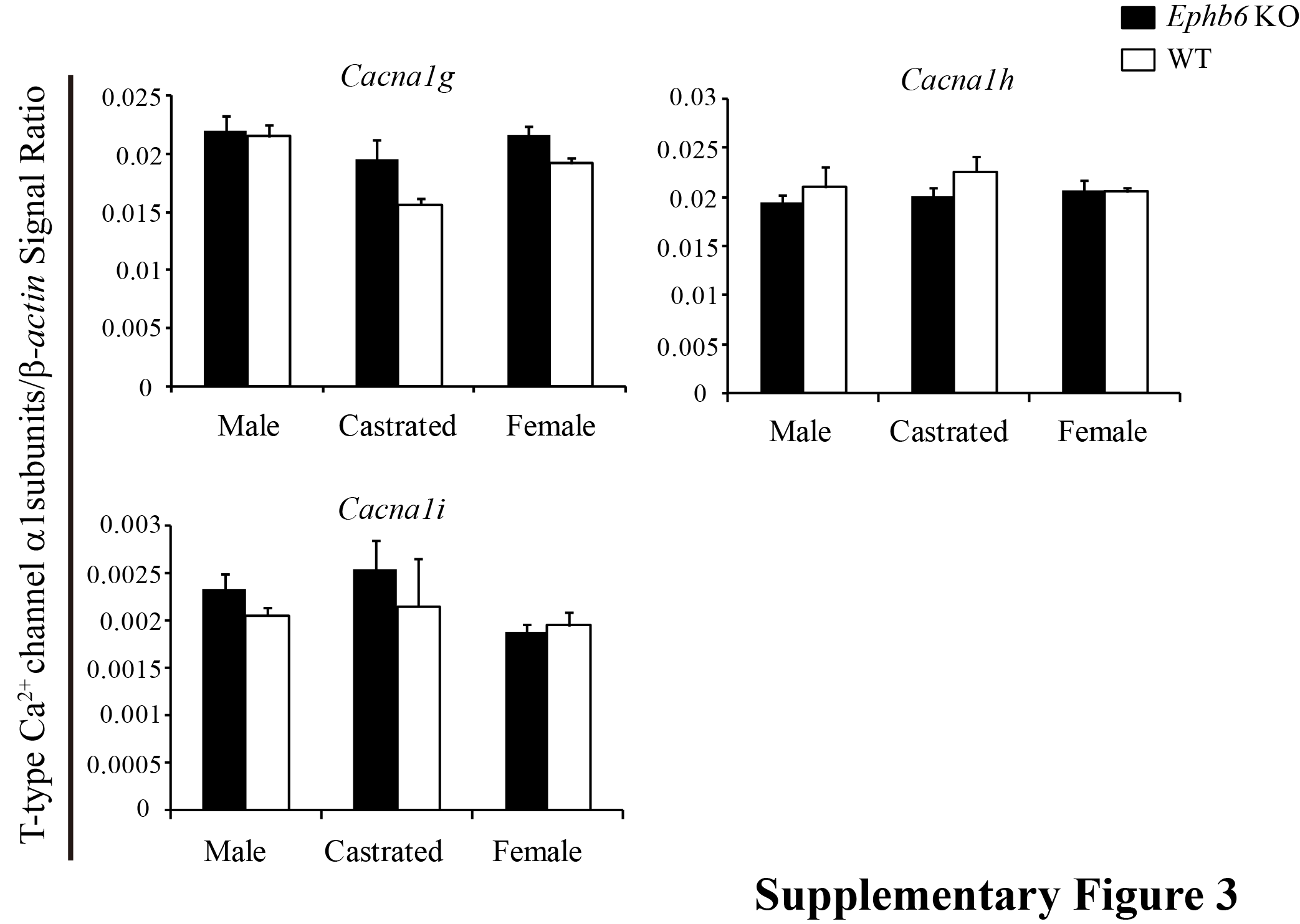
*

*Supplementary Figure 3. Normal mRNA expression of T-type Ca^2+^ channel a1 subunits in the adrenal gland medullae of male, female and castrated male EPHB6 KO mice*

Total RNA was extracted from adrenal gland medullae of male, female and castrated male WT and *EPHB6* KO mice. T-type Ca^2+^ channel α1 subunit *Cacna1g*, *Cacna1h* and *Cacna1i*mRNA levels in the tissues were measured by RT-qPCR. Experiments and data analysis were conducted as described in Supplementary Figure 1.


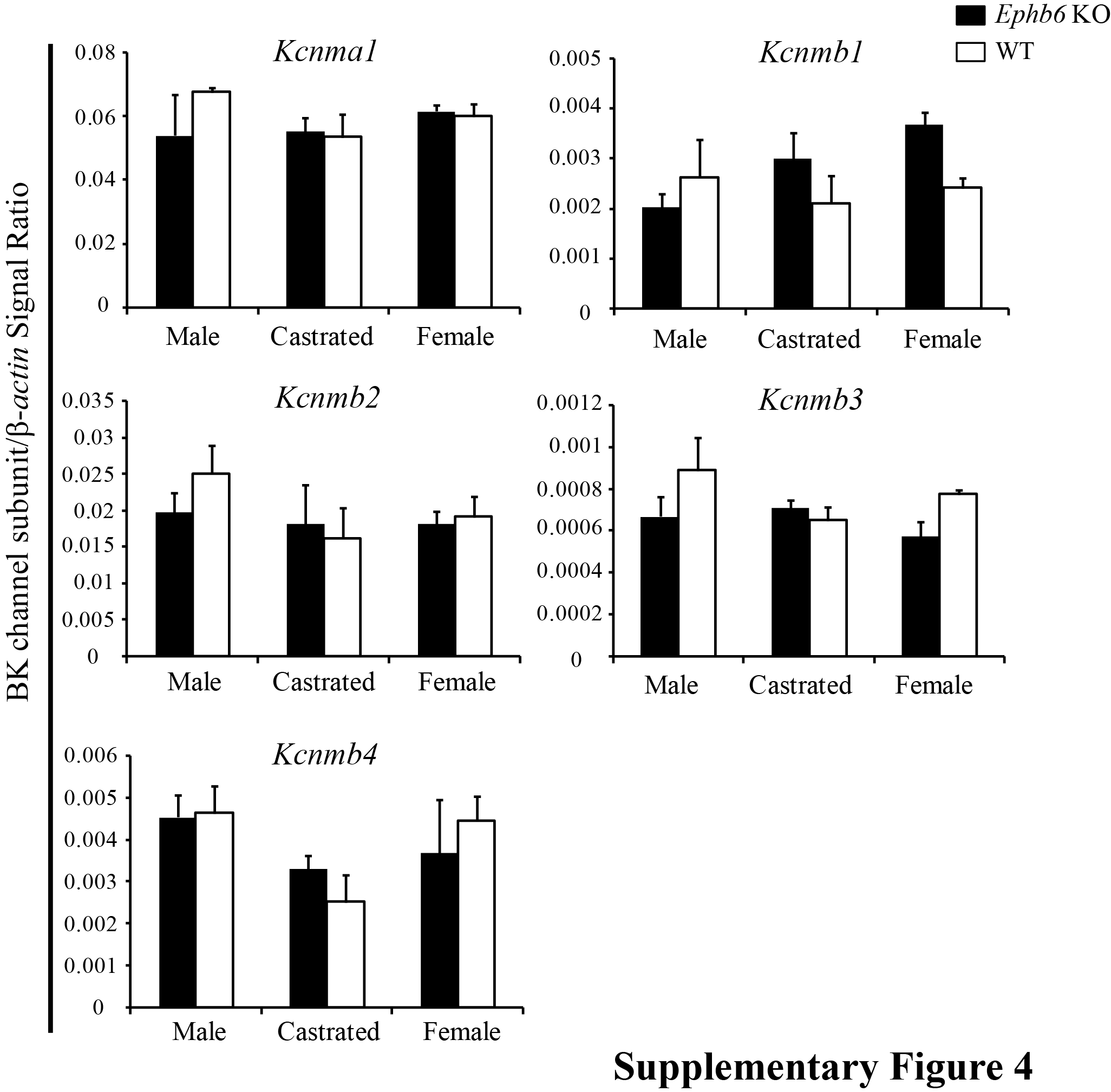


*Supplementary Figure 4. Normal mRNA expression of BK channel subunits in adrenal gland medullae of male, female and castrated male KO and WT mice*

Total RNA was extracted from adrenal gland medullae of WT and *Ephb6* KO male, female and castrated male mice. mRNA levels of the BK channel α subunit *Kcnma1* or the β-subunits *Kcnmb1, Kcnmb2, Kcnmb3 and Kcnmb4* in the medullae were measured by RT-qPCR. Experiments and data analysis were conducted as described in Supplementary Figure1.
